# Subjective mental fatigue ratings are not associated with basketball game-related statistics during official semiprofessional male games

**DOI:** 10.3389/fpsyg.2025.1644762

**Published:** 2025-08-18

**Authors:** Pierpaolo Sansone, Leonardo de Sousa Fortes, Damiano Polverari, Anthony Leicht, Miguel Angel Gomez-Ruano

**Affiliations:** ^1^Department of Education and Sport Sciences, Pegaso Telematic University, Naples, Italy; ^2^Department of Sports, Federal University of Minas Gerais, Belo Horizonte, Brazil; ^3^GranTorino Basketball Draft, Turin, Italy; ^4^Sport and Exercise Science, James Cook University, Townsville, QLD, Australia; ^5^Faculty of Physical Activities and Sport Sciences, Universidad Politécnica de Madrid, Madrid, Spain

**Keywords:** technical-tactical performance, cognitive, team sports, athlete monitoring, sport psychology, fatigue, athlete-reported outcome measures

## Abstract

**Introduction:**

Mental fatigue (MF) has been shown to affect several domains of team sports performance, including physical, technical, and perceptual-cognitive aspects. This study examined whether subjective ratings of mental fatigue (MF) are associated with technical-tactical performance in adult male basketball official games.

**Methods:**

Fourteen semiprofessional players (age: 22.1 ± 3.8 years) were monitored across 15 in-season weeks, encompassing 17 official games. The day before the game, players reported their level of MF using 100-mm visual analog scales. Technical-tactical performance during games was assessed by retrieving game-related statistics (GRS) (points; 2-point shots (2P) made; 2P missed; 2P percentage (2P%); 3-point (3P) shots made; 3P shots missed; 3P shot percentage (3P%); free-throws (1P) made; 1P missed; 1P percentage (1P%); offensive rebounds; defensive rebounds; assists; steals; turnovers; blocks; blocks against; fouls committed; fouls drawn; and Performance Index Rating). To control for games with different paces, games were coded as faster or slower according to the number of ball possessions. Separate linear mixed models evaluated the effects of variations in MF (z-scores) on GRS.

**Results:**

MF did not influence any GRS (all *p* > 0.05), while 3P misses were higher in faster games compared to slower (*p* = 0.049).

**Conclusions:**

Subjective ratings of MF are not associated with technical-tactical performance in male semiprofessional basketball. Current findings indicate that male semiprofessional basketball players are able to maintain technical-tactical performances despite the presence of MF.

## 1 Introduction

Basketball is a team sport in which two teams compete for victory. Sport science and performance analysts have studied the multiple domains of basketball performance, including the physical, physiological, perceptual-cognitive, and technical-tactical aspects, to identify which factors discriminate successful performances (Stojanovic et al., 2018; Çene, 2018; Fortes et al., 2022). While physical and physiological elements do not seem to discriminate successful teams within competitive standards (Sansone et al., 2025), many studies have identified that game-related statistics (GRS) (i.e., points, rebounds, turnovers, etc.) discriminate better team sport performances (Çene, 2018; Zhang et al., 2020) as well as more skilled players (Gasperi et al., 2020; Gomez et al., 2009; Mateus et al., 2020). Thus, GRS are considered one of the essential performance aspects that basketball performance staff must consider to optimize their players' and team's chances of success in competition.

Key performance indicators such as shooting statistics (i.e., shots made, accuracy), rebounding, assists, and turnovers depend on basketball fundamentals (i.e., perceptual-cognitive and technical skills) (Conte et al., 2019; Daub et al., 2023a,b), and on players' tactical abilities, which can be conceptualized as the ability of the player to appropriately act in the complex, dynamic, and non-linear scenarios emerging in basketball matches (García-Rubio et al., 2017; de Saá Guerra et al., 2013). For instance, to score, the basketball player needs to have a proper shooting form (i.e., technical skill) (Li et al., 2021) and undertake an appropriate decision on if/when to take a shot relevant to the specific game scenario (i.e., tactical ability) (Okazaki and Rodacki, 2012). Similar examples exist for other GRS. For instance, rebounding requires both a good technique for boxing out, jumping, and securing the ball (i.e., the skill) alongside assessment of the situation and court presence with timely planning of all actions. The dynamic and stochastic nature of basketball (de Saá Guerra et al., 2013; Stojanovic et al., 2018) implies that decision-making, perceptual-cognitive processes (i.e., attention, visual search behavior, and pattern recognition), and reactive abilities (i.e., players' tactical abilities) are elemental aspects of successful performance (Fortes et al., 2022; Kinrade et al., 2015). Accordingly, these performance determinants are well-understood by basketball coaches and performance staff, who regularly implement skills and tactical training (i.e., small-sided matches) (Bredt et al., 2021; Sansone et al., 2023) to appropriately prepare players for matches, aiming to increase the team's success.

While physical training preparation is important for basketball athletes, players must be prepared cognitively for competition. Suboptimal cognitive conditions, such as the presence of mental fatigue (MF), have been documented to impair key team sport performance aspects (Cutsem et al., 2017; Sun et al., 2021). MF is the psychobiological state caused by high cognitive effort from cognitive load (e.g., short-time with high complexity cognitive activity or prolonged periods with low complexity cognitive activity) that results in feelings of tiredness, lethargy, and lack of energy (Marcora et al., 2009; Smith et al., 2016), leading to early disengagement from physical performance and poor executive function (Smith et al., 2018). Further, MF-induced impairments of executive function can lead to poorer psychomotor performance (Habay et al., 2021), such as accuracy, response time, attention on task, action preparation, and planning (Smith et al., 2018; Habay et al., 2021). As these cognitive performance aspects underpin technical-tactical performance in basketball (i.e., fundamentals), monitoring MF alongside basketball GRS may assist with players' performances and, in turn, team success. Previously, MF was reported to impair several key aspects of basketball technical-tactical performance, including shooting (Filipas et al., 2021; Daub et al., 2023a,b), visuomotor (Faro et al., 2023) and decision-making skills (Fortes et al., 2022). However, other technical-tactical performance variables essential for basketball success, such as rebounding, assists, and fouls (Gomez et al., 2009; Zhang et al., 2020; Sansone et al., 2024), have not been investigated with MF. Importantly, while previous basketball studies have provided valuable information on the effects of MF on selected performance parameters, none have assessed technical-tactical parameters within the most important ecological setting, namely official matches. As recently suggested by Lam et al. (2025), it is essential to consider MF within ecological sport settings to better comprehend this interesting phenomenon. Therefore, this study aimed to examine the impact of MF on basketball technical-tactical performance (i.e., GRS) during official basketball matches. We hypothesize that higher MF levels will correspond with lower technical-tactical performances.

## 2 Materials and methods

### 2.1 Participants and study design

Fourteen adult male basketball players (age: 22.1 ± 3.8 years; height: 192.6 ± 8.8 cm; body mass: 85.5 ± 9.1 kg) voluntarily participated in this study. Players competed within an interregional basketball league (Tier 3 according to McKay et al., 2022) and typically participated in 5 team-based basketball training sessions, 2–4 individual physical training sessions, and 1–2 official matches per week (Tier 3) (McKay et al., 2022). Each player was informed about the study procedures and provided informed, written consent under approval by the Institutional Review Board of the Pegaso Telematic University (#001218).

It was an observational study with a longitudinal design (repeated measures). Players were monitored for 15 consecutive weeks during the 2024–2025 basketball season, encompassing 17 official matches. Data from players who had < 5 min of playing time in single games were excluded (Gomez et al., 2009), which led to a total of 119 observations (50% of possible observations).

### 2.2 Monitoring of subjective mental fatigue

Before the data collection, an experienced sport scientist explained the definition of MF psychobiological state induced by high or prolonged cognitive effort characterized by feelings of tiredness, lethargy, and lack of energy to facilitate players' understanding and avoid confusion throughout the study (Marcora et al., 2009; Smith et al., 2016).

Subjective MF ratings were collected the day before official games (i.e., MD-1), which was typically a rest day scheduled at the end of the training week. MD-1 was preferred to reduce the risk of inaccurate reporting by athletes, as previous evidence suggests they can lie due to concerns about potential misinterpretations of subjective data and reduced sport opportunities (Coventry et al., 2023) (i.e., reductions in playing time). Players reported their MF using 100-mm visual-analog scales, with two anchors (0: no mental fatigue; 100: maximal mental fatigue). This method has been demonstrated to be valid and practical (Smith et al., 2019) for registering subjective MF ratings and used in relevant team sports research (Daub et al., 2023a,b; Díaz-García et al., 2023). For each athlete, MF Z-scores (MF-Z) were calculated as follows: (today's individual player score—individual player's average)/individual player's standard deviation (SD). The *Z*-score represents the number of SDs the player's MF rating was above or below the mean of the distribution (Gallo et al., 2016). When collecting athlete-reported outcome measures, *Z*-score calculation allows an understanding of the relative change in response for each player (Ryan et al., 2018), thus providing accurate information for each individual and limiting potential confounding effects related to the interpretation and use of the subjective scale used.

### 2.3 Game-related statistics

For each game, the following individual GRS were retrieved from the official league statistics website: points; 2-point shots (2P) made; 2P missed; 2P percentage (2P%); 3-point shots made; 3-points shots missed; 3-point shot percentage (3P%); free-throws (1P) made; 1P missed; 1P percentage (1P%); offensive rebounds; defensive rebounds; assists; steals; turnovers; blocks; blocks against; fouls committed; fouls drawn; and Performance Index Rating (PIR, calculated as [points + rebounds + assists + steals + blocks + fouls drawn]—[missed field goals + 1P missed + turnovers + blocks against + fouls committed]). These variables were selected as the prominent technical-tactical performance indicators used in basketball research (Sansone et al., 2024; Zhang et al., 2017; Gómez et al., 2008) and practice. To control for match exposure, GRS were normalized by the individual playing time (e.g., a player scoring 10 points over 20 min of playing time = 0.5 points per minute) (Sansone et al., 2024; Gomez et al., 2009).

To control for the effect of game pace (Chen et al., 2025), the number of team ball possessions was quantified using the following established formula (Charamis et al., 2023): possessions = field goals attempted – offensive rebounds + turnovers + 0.454 × free throws attempted. Then, k-means cluster analysis was used to classify games as fast (cluster center: 87.5 possessions) or slow (cluster center: 78.9 possessions) paced (Chen et al., 2025).

### 2.4 Statistical analyses

Statistical analyses were performed using Jamovi software (version 2.3; https://www.jamovi.org/), with the α level set at 0.05. Visual inspection of all data was performed to assess distribution and outliers. The variable block against was excluded due to an excessive number of outliers. Separate linear mixed models (one for each GRS) were performed to evaluate if technical-tactical performances were influenced by MF-Z or game pace (fixed effects), while players' ID was inserted as a random effect to account for repeated measures. For each model, Akaike's Information Criterion (AIC), *F-*, and *P*-values were reported.

## 3 Results

**Figure 1:**
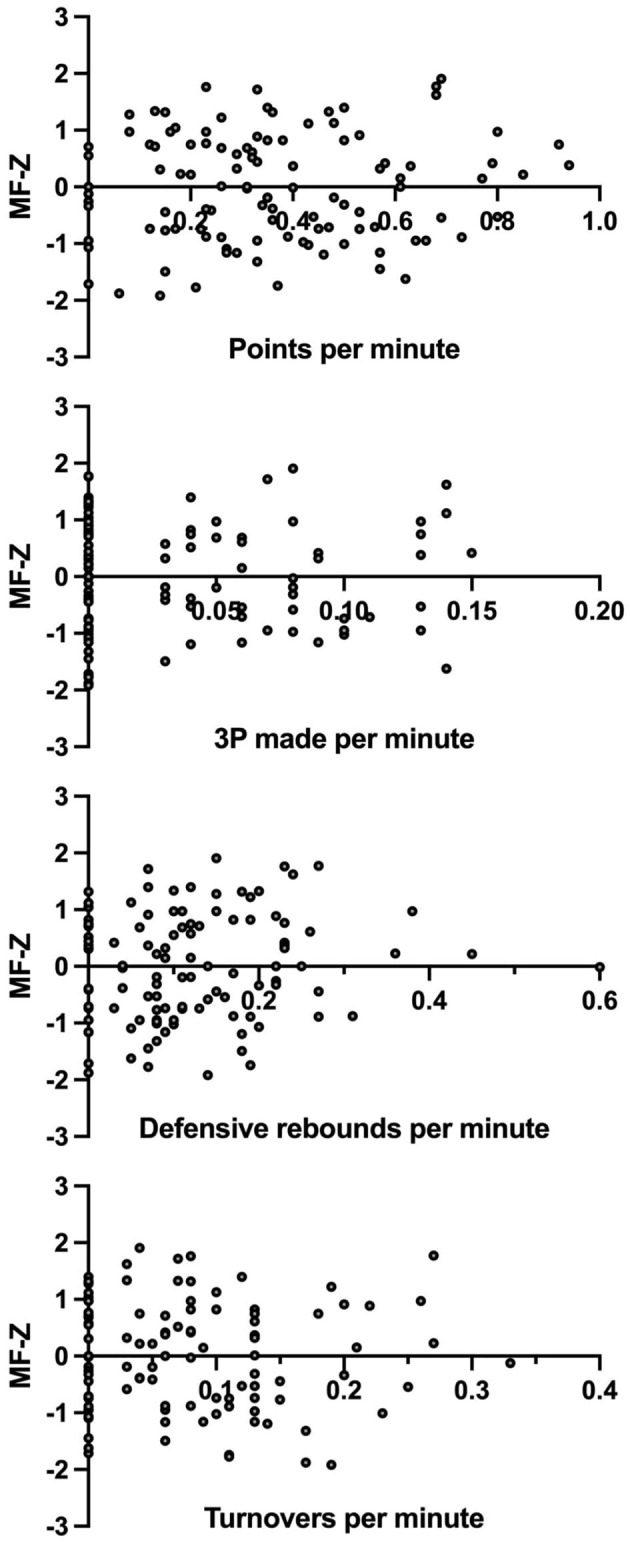
Data of selected game-related statistics according to variations in mental fatigue.

Table 1 reports the descriptive statistics for all GRS during the season. Table 2 reports the AIC, F, and *p*-values for all models. There were no significant effects of MF-Z on any GRS (all *p* >0.05, Figure 1 for a sample of GRS). Regarding game pace, only 3P missed were influenced with higher values during fast compared to slow matches (0.10 ± 0.07 vs. 0.07 ± 0.05; *p* = 0.049).

**Table 1:** Descriptive data for the game-related statistics.

**Performance indicator**	**Mean and standard deviation**
Points	0.36 ± 0.23
2P made	0.10 ± 0.08
2P missed	0.11 ± 0.07
2P%	46.29 ± 29.35
3P made	0.03 ± 0.04
3P missed	0.09 ± 0.10
3P%	27.70 ± 28.54
1P made	0.06 ± 0.07
1P missed	0.03 ± 0.05
1P%	66.76 ± 28.27
Offensive rebounds	0.07± 0.09
Defensive rebounds	0.12 ± 0.11
Assists	0.06 ± 0.07
Steals	0.03 ± 0.03
Turnovers	0.08 ± 0.08
Blocks	0.02 ± 0.04
Fouls committed	0.12 ± 0.10
Fouls drawn	0.08 ± 0.09
PIR	0.31 ± 0.38

All game-related statistics are normalized per minute of playing time.

**Table 2:** Results of the linear mixed models.

**Performance indicator**	**AIC**	**MF-Z**	**Game pace**
Points	−12.31690	*F* = 0.807, *p* = 0.371	*F* = 0.112, *p* = 0.738
2P made	−225.88520	*F* = 0.8492, *p* = 0.359	*F* = 0.0586, *p* = 0.809
2P missed	−255.1336	*F* = 0.480, *p* = 0.490	*F* = 1.860, *p* =0.176
2P%	1015.9154	*F* = 0.00947, *p* = 0.923	*F* = 1.49946, *p* = 0.224
3P made	−370.051	*F* = 0.0802, *p* = 0.778	*F* = 0.0473, *p* = 0.828
3P missed	−214.2078	*F* = 2.34, *p* = 0.130	***F*** **=** **3.96**, ***p*** **=** **0.049**
3P%	684.0053	*F* = 0.888, *p* = 0.349	*F* = 1.476, *p* = 0.229
1P made	−264.91580	*F* = 0.0157, *p* = 0.901	*F* = 0.2909, *p* = 0.591
1P missed	−342.4608	*F* = 1.277, *p* = 0.261	*F* = 0.505, *p* = 0.479
1P%	635.9662	*F* = 0.490, *p* = 0.486	*F* = 0.691, *p* = 0.409
Offensive rebounds	−239.10620	*F* = 0.452, *p* = 0.514	*F* = 0.005, *p* = 0.949
Defensive rebounds	−190.7931	*F* = 1.97, *p* = 0.164	*F* = 3.51, *p* = 0.064
Assists	−274.67130	*F* = 1.4970, *p* = 0.224	*F* = 0.0126, *p* = 0.911
Steals	−404.41450	*F* = 0.0457, *p* = 0.831	*F* = 0.8569, *p* = 0.357
Turnovers	−248.5562	*F* = 0.0871, *p* = 0.769	*F* = 2.2687, *p* = 0.135
Blocks	−369.35840	*F* = 0.1759, *p* = 0.846	*F* = 0.1759, *p* = 0.676
Fouls committed	−188.7038	*F* = 2.0349, *p* = 0.157	*F* = 0.0671, *p* = 0.796
Fouls drawn	−232.602	*F* = 0.002, *p* = 0.966	*F* = 0.145, *p* = 0.704
PIR	99.9351	*F* = 1.5086, *p* = 0.222	*F* = 0.0293, *p* = 0.864

Significant effects in bold.

## 4 Discussions

This study was the first to assess whether MF affects basketball technical-tactical performance during official games. Contrary to our hypothesis, current findings indicate that, in adult male semi-professional players, subjective MF ratings collected the day before the game were not associated with GRS in the game played the subsequent day. Therefore, it appears that, in ecological settings, subjective MF does not affect technical-tactical performance in basketball, which should be considered by basketball practitioners monitoring the player's status by means of athlete-reported outcome measures.

To date, the negative effects of MF on sports performance have been reported predominantly within controlled, experimental test settings (Habay et al., 2021) where performance was evaluated following the exposure to MF-inducing protocols (e.g., often a 30-min Stroop test). In such studies, MF was reported to negatively impact technical-tactical performance, including decision-making, accuracy, passing, and shooting for basketball athletes (Fortes et al., 2022; Filipas et al., 2021; Smith et al., 2016). While these performance domains underpin the GRS monitored in the current study, no association between subjective MF and performance was found. Our results agree with a recent study reporting that subjective MF ratings were not associated with technical-tactical performance in Australian Football matches (Joseph et al., 2025). A potential explanation for the lack of association between subjective MF and GRS in the current study, as well as in recent findings from Australian Football (Joseph et al., 2025), relates to the different approaches used to assess MF. Experimental studies typically induce MF acutely through controlled cognitive tasks (e.g., Stroop tests), often resulting in marked impairments in performance (Fortes et al., 2022; Filipas et al., 2021; Smith et al., 2016). In contrast, descriptive studies conducted in ecological settings (i.e., across the competitive season) have monitored MF using subjective questionnaires (Díaz-García et al., 2023; Russell et al., 2020; Thompson et al., 2020), which might capture more chronic and variable levels of MF. These differences in methodology may partly explain the inconsistent results. Further exploring the temporal nature of MF, this phenomenon occurs naturally in sports settings (Lam et al., 2025) and can manifest acutely (i.e., after a specific task or exertion), cumulatively (i.e., evolving during the competitive season), or both (Russell et al., 2022, 2019; André et al., 2025). In fact, recent relevant studies (Russell et al., 2019; Lam et al., 2025) have emphasized the importance of considering the cumulative nature of MF as this is a key feature of how athletes experience it. When considering our direct findings and those of Joseph et al. (2025), and following what André et al. (2025) indicated, it can be hypothesized that athletes develop a degree of tolerance or adaptation to MF, intended as a reduction in their mental fatigability or MF-susceptibility (André et al., 2025), across the competitive season (André et al., 2025) which allows them to maintain performances despite the presence of subjective MF.

In fact, it is important to acknowledge here that increased motivation, willpower and the perceived value of the reward from the activity can counteract the negative effects of MF on performance (Schiphof-Godart et al., 2018). These aspects seem highly relevant for team sports seen their complex nature, with athletes' performances being determined by the non-linear interaction between physical, physiological, technical-tactical, psychological and contextual factors (Stojanovic et al., 2018; Robazza et al., 2012; Sansone et al., 2025). It is thus plausible to hypothesize that MF might affect performances differently on specific days and phases of the competitive season. In a previous study, professional basketball players reported the highest levels of motivation on the game day (Clemente et al., 2019), obviously stimulated by the importance and excitement induced by the competitive setting. Along this line, the same could happen in decisive season phases such as playoffs, play-outs and finals in which the increased importance of games can benefit motivation and willpower, thus counteract potential negative consequences of MF. While previous studies have reported higher subjective MF during congested schedules (Thompson et al., 2020) and in critical phases of the season such as playoffs (Díaz-García et al., 2023), these studies did not measure any performance outcome. Seen the potential interaction of schedule, season phase, MF and performance, we recommend future team sport studies to monitor these aspects concurrently.

In the current study, subjective MF was assessed the day before the match (MD-1) which is typically characterized by substantial reductions of training load (Clemente et al., 2019; Manzi et al., 2010) and thus may not capture acute or peak levels of fatigue. Differently, in experimental studies MF is induced immediately before performance testing. While the temporal gap between MF assessment and the game in this study may have limited the sensitivity to detect such effects, the game day is typically associated with higher motivation and improved wellbeing in basketball (Clemente et al., 2019). Thus, it is plausible that, in the current study, players were cognitively prepared, motivated by the upcoming competition and sufficiently rested, which led to the absence of any negative effect of subjective MF on technical-tactical performance. Seen that team sport athletes can differentiate MF from subjective feelings of mood, stress and motivation (Russell et al., 2022), these factors may contribute to the absence of a relationship between subjective MF and performance in this study.

Regarding the game pace, more 3P were missed in fast-paced games. Such games are characterized by a higher number of ball possessions (Charamis et al., 2023), which leads to more shots registered, including three-pointers. These 3P shots might be especially taken in transition offense, a scenario that is more frequent in faster-paced games compared to slower ones (Chen et al., 2025), in order to take advantage of potential imbalances in the opponent's defense.

This study has indicated that MF had no impact on GRS in semi-professional basketball players during a full season, indicating well-prepared athletes. However, some limitations exist, including the monitoring of a single team within a semi-professional competition, the data collection scheduled on the day before competition, and only in male settings. Future research on mental fatigue (MF) and basketball performance should monitor other samples (i.e., elite players, women), measure MF levels right before competition, and aim to provide actionable insights for coaches and practitioners by incorporating objective MF markers (e.g., brain-based measures) (Roelands and Jeroen, 2022). Additionally contextual factors (e.g., game schedule, academic demands) should be taken in consideration alongside the assessment of additional perceptual-cognitive variables potentially linked to game-related statistics (such as motivation and mood). This approach may enhance support strategies to better prepare athletes for competition despite fluctuating cognitive states.

## 5 Conclusions

The current study shows that, in adult semiprofessional male basketball, subjective mental fatigue ratings collected the day before competition were not associated with technical-tactical performance, as measured by game-related statistics. These findings suggest that, under typical in-season conditions, players may be able to maintain performance despite varying levels of mental fatigue—an important consideration for coaches and support staff when interpreting athlete readiness and planning interventions.

## Data Availability

The raw data supporting the conclusions of this article will be made available by the authors, without undue reservation.

## References

[B1] André N. Audiffren M. Englert C. (2025). Brain endurance training as a strategy for reducing mental fatigue. Front. Psychol. 16:1616171. 10.3389/fpsyg.2025.1616171 40606889 PMC12213578

[B2] Bredt S. G. T. Camargo D. S. Mortoza B. V. B. Pereira de Andrade A. G. Paolucci L. A. Rosso T. L. N.. (2021). Additional players and half-court areas enhance group tactical-technical behavior and decrease physical and physiological responses in basketball small-sided games. Int. J. Sports Sci. Coach. 17, 1079–1088. 10.1177/17479541211053638

[B3] Çene E. (2018). What is the difference between a winning and a losing team: insights from Euroleague basketball. Int. J. Perform. Anal. Sport 18, 55–68. 10.1080/24748668.2018.1446234

[B4] Charamis E. Marmarinos C. Ntzoufras I. (2023). Estimating team possessions in high-level european basketball competition. Int. J. Sports Sci. Coach. 18, 220–230. 10.1177/17479541211070788

[B5] Chen R. Zhang M. Xu X. Liu Y. (2025). Game-related statistics for distinguishing winning and losing teams in olympic basketball: the impact of game pace. J. Sports Sci. 42, 2541–2552. 10.1080/02640414.2024.2448360 39742428

[B6] Clemente F. M. Mendes B. Bredt D. G. T. Praça G. M. Silvério A. Carriço S.. (2019). Perceived training load, muscle soreness, stress, fatigue, and sleep quality in professional basketball: a full season study. J. Hum. Kinet. 67, 199–207. 10.2478/hukin-2019-0002 31523318 PMC6714361

[B7] Conte D. Smith M. R. Santolamazza F. Favero T. G. Tessitore A. Coutts A. (2019). Reliability, usefulness and construct validity of the combined basketball skill test (CBST). J. Sports Sci. 11, 1205–1211. 10.1080/02640414.2018.1551046 30499758

[B8] Coventry M. Timler A. Mosler A. B. Russell K. Travers M. Mitchell L.. (2023). ‘I lied a little bit.' A qualitative study exploring the perspectives of elite Australian athletes on self-reported data. Phys. Ther. Sport 60, 91–97. 10.1016/j.ptsp.2023.01.009 36738670

[B9] Cutsem J. V. Marcora S. De Pauw K. Bailey S. Meeusen R. Roelands B. (2017). The effects of mental fatigue on physical performance : a systematic review. Sports Med. 47, 1569–1588. 10.1007/s40279-016-0672-0 28044281

[B10] Daub B. D. McLean B. D. Heishman A. D. Coutts A. J. (2023a). The reliability and usefulness of a novel basketball standardized shooting task. Int. J. Sports Sci. Coach. 18, 1285–1294. 10.1177/17479541221100496

[B11] Daub B. D. Mclean B. D. Heishman A. D. Peak K. M. Coutts A. J. (2023b). The relationship between mental fatigue and shooting performance over the course of a national collegiate athletic association division i basketball season. J. Strength Condit. Res. 38, 334–341. 10.1519/JSC.0000000000004624 38090974

[B12] de Saá Guerra Y. Gonzalez J. M. M. Montesdeoca S. S. Ruiz D. R. López N. A. García-Manso J. M. (2013). Basketball scoring in NBA games: an example of complexity. J. Syst. Sci. Complexity 26, 94–103. 10.1007/s11424-013-2282-3

[B13] Díaz-García J. Filipas L. La Torre A. Gómez-Rivera J. Rubio-Morales A. García-Calvo T. (2023). Mental fatigue changes from regular season to play-offs in semiprofessional soccer: a comparison by training days. Scand. J. Med. Sci. Sports 33, 712–724. 10.1111/sms.14301 36601789

[B14] Faro H. de Sousa F. L. Dalton L.-J. Teixeira B. B. Caputo F. M. E. Sousa S.. (2023). Sport-based video game causes mental fatigue and impairs visuomotor skill in male basketball players. Int. J. Sport Exerc. Psychol. 21, 1125–1139. 10.1080/1612197X.2022.2109187

[B15] Filipas L. Ferioli D. Banfi G. La Torre A. Vitale J. A. (2021). Single and combined effect of acute sleep restriction and mental fatigue on basketball free-throw performance. Int. J. Sports Physiol. Perform. 16, 415–420. 10.1123/ijspp.2020-0142 33440343

[B16] Fortes L. S. Lima-Junior D. Barbosa B. T. Faro H. K. C. Ferreira M. E. C. Almeida S. S. (2022). Effect of mental fatigue on decision-making skill and visual search behaviour in basketball players: an experimental and randomised study. Int. J. Sport Exerc. Psychol. 1–20. 10.1080/1612197X.2022.2058055

[B17] Gallo T. F. Cormack S. J. Gabbett T. J. Lorenzen C. H. (2016). Pre-training perceived wellness impacts training output in australian football players. J. Sports Sci. 34, 1445–1451. 10.1080/02640414.2015.1119295 26637525

[B18] García-Rubio J. Gómez M. A. Cañadas M. Ibáñez S. J. (2017). Offensive rating-time coordination dynamics in basketball. Complex systems theory applied to basketball. Int. J. Perform. Anal. Sport 15, 513–526. 10.1080/24748668.2015.11868810

[B19] Gasperi L. Conte D. Leicht A. Gómez-Ruano M. A. (2020). Game related statistics discriminate national and foreign players according to playing position and team ability in the women's basketball Euroleague. Int. J. Environ. Res. Public Health 17, 1–10. 10.3390/ijerph17155507 32751559 PMC7432202

[B20] Gómez M. A. Lorenzo A. Barakat R. Ortega E. José J. M. (2008). Differences in game-related statistics of basketball performance by game location for men's winning and losing teams. Percept. Mot. Skills 106, 43–50. 10.2466/pms.106.1.43-50 18459354

[B21] Gomez M. A. Lorenzo A. Ortega E. Sampaio J. E. Ibañez S. (2009). Game related statistics discriminating between starters and nonstarters players in Women's National Basketball Association League (WNBA). J. Sports Sci. Med. 8, 278–283. 24149538 PMC3761479

[B22] Habay J. Van Cutsem J. Verschueren J. De Bock S. Proost M. De Wachter J.. (2021). Mental fatigue and sport-specific psychomotor performance: a systematic review. Sports Med. 51, 1527–1548. 10.1007/s40279-021-01429-6 33710524

[B23] Joseph S. D. Russell S. Halson S. L. Johnston R. D. Timmins R. G. Murray N. B.. (2025). Mental fatigue, skill performance and activity profile in elite male australian football match play. J. Sports Sci. 43, 1185–1195. 10.1080/02640414.2025.2489883 40214014

[B24] Kinrade N. P. Jackson R. C. Ashford K. J. (2015). Reinvestment, task complexity and decision making under pressure inbasketball. Psychol. Sport Exerc. 20, 11–19. 10.1016/j.psychsport.2015.03.007

[B25] Lam H. K. N. Sproule J. Phillips S. M. (2025). Future directions in understanding acute and chronic effects of mental fatigue in sports: a commentary on bridging laboratory findings and real-world applications. Int. J. Sports Physiol. Perform. 20, 1172–1176. 10.1123/ijspp.2024-0363 39837313

[B26] Li F. Li Z. Borović I. Rupčić T. Knjaz D. (2021). Does fatigue affect the kinematics of shooting in female basketball? Int. J. Perform. Anal. Sport 21, 754–766. 10.1080/24748668.2021.1945878

[B27] Manzi V. D'Ottavio S. Impellizzeri F. M. Chaouachi A. Chamari K. Castagna C. (2010). Profile of weekly training load in elite male professional basketball players. J. Strength Condit. Res. 24, 1399–1406. 10.1519/JSC.0b013e3181d7552a 20386474

[B28] Marcora S. M. Staiano W. Manning V. (2009). Mental fatigue impairs physical performance in humans. J. Appl. Physiol. 106, 857–864. 10.1152/japplphysiol.91324.2008 19131473

[B29] Mateus N. Esteves P. Gonçalves B. Torres I. Gomez M. A. Arede J.. (2020). Clustering performance in the european basketball according to players' characteristics and contextual variables. Int. J. Sports Sci. Coach. 15, 405–411. 10.1177/1747954120911308

[B30] McKay A. K. A. Stellingwerff T. Smith E. S. Martin D. T. Mujika I. Goosey-Tolfrey V.-L.. (2022). Defining training and performance caliber: a participant classification framework. Int. J. Sports Physiol. Perform. 17, 317–331. 10.1123/ijspp.2021-0451 34965513

[B31] Okazaki V. H. A. Rodacki A. L. F. (2012). Increased distance of shooting on basketball jump shot. J. Sports Sci. Med. 11, 231–237. 24149195 PMC3737873

[B32] Robazza C. Gallina S. D'Amico M. A. Izzicupo P. Bascelli A. Di Fonso A.. (2012). Relationship between biological markers and psychological states in elite basketball players across a competitive season. Psychol. Sport Exerc. 13, 509–517. 10.1016/j.psychsport.2012.02.011

[B33] Roelands B. Jeroen V. C. (2022). Brain research into the mechanisms and consequences of mental fatigue. Perform. Enhanc. Health 10:100239. 10.1016/j.peh.2022.100239

[B34] Russell S. Jenkins D. Halson S. Kelly V. (2020). Changes in subjective mental and physical fatigue during netball games in elite development athletes. J. Sci. Med. Sport 23, 615–620. 10.1016/j.jsams.2019.12.017 31883778

[B35] Russell S. Jenkins D. Rynne S. Halson S. L. Kelly V. (2019). What is mental fatigue in elite sport? Perceptions from athletes and staff. Eur. J. Sport Sci. 19, 1367–1376. 10.1080/17461391.2019.1618397 31081474

[B36] Russell S. Jenkins D. G. Halson S. L. Juliff L. E. Connick M. J. Kelly V. G. (2022). Mental fatigue over 2 elite netball seasons: a case for mental fatigue to be included in athlete self-report measures. Int. J. Sports Physiol. Perform. 17, 160–169. 10.1123/ijspp.2021-0028 34583327

[B37] Ryan S. Coutts A. J. Hocking J. Dillon P. A. Whitty A. Kempton T. (2018). Physical preparation factors that Infl uence technical and physical match performance in professional Australian Football 13, 1021–1027. 10.1123/ijspp.2017-0640 29466065

[B38] Sansone P. Conte D. Gasperi L. Scanlan A. T. Gomez-Ruano M. A. (2024). Analysing elite European basketball players' performances according to travel demands, game schedule and contextual factors. Int. J. Perform. Anal. Sport 25, 446–461. 10.1080/24748668.2024.2418185

[B39] Sansone P. Gasperi L. Makivic B. Gomez-Ruano M. A. Tessitore A. Conte D. (2023). An ecological investigation of average and peak external load intensities of basketball skills and game-based training drills. Biol. Sport 40, 649–656. 10.5114/biolsport.2023.119291 37398975 PMC10286615

[B40] Sansone P. Perez-Chao E. Li F. Gasperi L. Gomez-Ruano M. A. Conte D. (2025). Contextual factors influencing basketball training and competition demands: a systematic review. Int. J. Sports Med. 46, 430–436. 10.1055/a-2533-0917 40090325

[B41] Schiphof-Godart L. Roelands B. Hettinga F. J. (2018). Drive in sports: how mental fatigue affects endurance performance. Front. Psychol. 9:1383. 10.3389/fpsyg.2018.01383 30174627 PMC6107844

[B42] Smith M. R. Chai R. Nguyen H. T. Marcora S. Coutts A. J. (2019). Comparing the effects of three cognitive tasks on indicators of mental fatigue. J. Psychol. 153, 759–783. 10.1080/00223980.2019.1611530 31188721

[B43] Smith M. R. Coutts A. J. Merlini M. Deprez D. Lenoir M. Marcora S. M. (2016). Mental fatigue impairs soccer-specific physical and technical performance. Med. Sci. Sports Exerc. 48, 267–276. 10.1249/MSS.0000000000000762 26312616

[B44] Smith M. R. Thompson C. Marcora S. M. Skorski S. Meyer T. Coutts A. J. (2018). Mental fatigue and soccer: current knowledge and future directions. Sports Med. 48, 1525–1523. 10.1007/s40279-018-0908-2 29623604

[B45] Stojanovic E. Stojiljkovic N. Scanlan A. T. Dalbo V. J. Berkelmans D. Milanovic Z. (2018). The activity demands and physiological responses encountered during basketball match-play : a systematic review. Sports Med. 48, 111–135. 10.1007/s40279-017-0794-z 29039018

[B46] Sun H. Soh K. G. Roslan S. Wazir M. R. W. N. Soh K. L. (2021). Does mental fatigue affect skilled performance in athletes? A systematic review. PLoS ONE 16:e0258307. 10.1371/journal.pone.0258307 34648555 PMC8516214

[B47] Thompson C. J. Noon M. Towlson C. Perry J. Coutts A. J. Harper L. D.. (2020). Understanding the presence of mental fatigue in english academy soccer players. J. Sports Sci. 38, 1524–1530. 10.1080/02640414.2020.1746597 32212903

[B48] Zhang S. Gomez M. A. Yi Q. Dong R. Leicht A. Lorenzo A. (2020). Modelling the relationship between match outcome and match performances during the 2019. FIBA basketball world cup: a quantile regression analysis. Int. J. Environ. Res. Public Health 17, 1–11. 10.3390/ijerph17165722 32784740 PMC7460061

[B49] Zhang S. Lorenzo A. Gómez M. A. Liu H. Gonçalves B. Sampaio J. (2017). Players' technical and physical performance profiles and game-to-game variation in Nba. Int. J. Perform. Anal. Sport 17, 466–483. 10.1080/24748668.2017.1352432

